# 16S rRNA sequence data of *Bombyx mori* gut bacteriome after spermidine supplementation

**DOI:** 10.1186/s13104-020-04958-x

**Published:** 2020-02-24

**Authors:** Resma Rajan, Alekhya Rani Chunduri, Anugata Lima, Anitha Mamillapalli

**Affiliations:** Department of Biotechnology, Institute of Science, GITAM (Deemed to be University), Visakhapatnam, 530 045 India

**Keywords:** *Bombyx mori*, Spermidine, Gut, Microbiome, Metagenome

## Abstract

**Objectives:**

The silkworm *Bombyx mori (B. mori)* is an important domesticated lepidopteran model for basic and applied research. They produce silk fibres that have great economic value. The gut microbiome plays an important role in the growth of organisms. Spermidine (Spd) is shown to be important for the growth of all living cells. The effect of spermidine feeding on the gut microbiome of 5th instar *B. mori* larvae was checked. The *B. mori* gut samples from control and spermidine fed larvae were subjected to next-generation sequencing analysis to unravel changes in the bacterial community upon spermidine supplementation.

**Data description:**

The changes in gut bacteriota after spermidine feeding is not studied before. *B. mori* larvae were divided into two groups of 50 worms each and were fed with normal mulberry leaves and mulberry leaves fortified with 50 µM spermidine. The gut tissues were isolated aseptically and total genomic DNA was extracted, 16S rRNA region amplified and sequenced using Illumina platform. The spermidine fed gut samples were shown to have abundance and diversity of the phyla *Proteobacteria, Firmicutes, Bacteroidetes,* and *Actinobacteria.*

## Objective

*B. mori* is an economically important and domesticated silkworm from family *Bombycidae* [1, 2]*. B. mori* feeds on mulberry leaves and produces silk which has been used as a fabric, biomaterial, and in cosmetics [3, 4]. The quality and quantity of the silk fibres produced depend on the food consumed [5]. The growth, absorption, and utilization of nutrients are influenced by the gut microbiota [6–8]. Bacterial flora associated with *B. mori* gut help them in degrading various otherwise inaccessible polysaccharides from the diet [9]. These complex communities are essential for the environmental adaptation and development of the host [10–15]. Polyamines are biogenic amines found in all eukaryotic cells, which perform distinct cellular functions [16]. Spd is a biologically important polyamine that serves as a key regulator of processes like DNA stability, protein synthesis, cell proliferation, differentiation, and apoptosis [17–19]. The potential effect of Spd supplementation to *B. mori* efficiently enhanced the larval weights, silk gland weights, silk quality and quantity, and mechanical and structural properties of the silk fibres [20, 21].

We aimed to understand changes in the gut microbiota post Spd supplementation when compared to control. The role of polyamines in biological functions, diseases, drug targeting, growth, free radical scavenging and cell viability are known. Our data could be of potential support to the researchers to understand the effect of Spd from a different perspective on gut bacteriota and foster a challenging and exciting research further. This study offers an improved diet with enhancement in beneficial microbes of the gut. This data was collected as part of finding the influence of Spd on the nutrition of *B. mori*. The detailed influence of Spd on the nutrition of *B. mori* 5th instar silkworms is unpublished research data.

## Data description

### Silk worm rearing and DNA isolation

The high silk yielding bivoltine breed, CSR2 × CSR4 *B. mori* larvae in 4th moult were procured from Andhra Pradesh state sericulture farm. Larvae were reared in a cleaned and disinfected room at 26 ± 2 °C with relative humidity of 65–85%. 50 µM Spd solution was applied to the mulberry leaves based on established protocol [21] from day 1 of 5th instar stage, and continued till the pupation (about 6 days) as during this stage larvae consume high quantity of mulberry leaf and produce maximum silk. The whole gut was extracted aseptically on day 5 and gut contents were flushed out for total genomic DNA isolation from both control and Spd fed groups (n = 1) using the QIAamp DNA Kit. The concentration and purity were determined by spectrophotometry.

### Library preparation and 16S rRNA sequencing

The 16S rRNA gene V3–V4 hypervariable regions were amplified using region-specific primers from KAPA HiFi HotStart PCR Kit (KAPA Biosystems Inc., Boston, MA USA). A negative control was maintained without the template DNA. A second-round indexing PCR was performed with the multiplexed amplicons which were amplified for 10 cycles with Illumina sequencing barcoded adaptors using Nextera XT v2 Index Kit, Illumina, U.S.A. The PCR products were analysed on 1.2% agarose gel after each round. The normalized, pooled and quantified libraries were used for Illumina MiSeq sequencing. 5% PhiX was spiked into introduce nucleotide diversity. The metagenome sequencing was carried out at Genotypic Technologies Pvt. Ltd., Bangalore, India.

### Analysis of metagenome sequence data

The Illumina paired-end reads were demultiplexed using bcl2fastqtool (“bcl2fastq” n.d.) and quality checked by FastQC [22]. The high- quality raw reads were stitched using Fastq-join [23] and further analysed using QIIMEpipeline [24]. The query sequences were clustered using the UCLUST5 method [25] against a curated chimera free 16S rRNA database (Greengenes v 13.8) [26]. The taxonomies were assigned using the RDP classifier [27] to these clusters at ≥ 50% sequence similarity against the reference database and generated of a biom file for further advanced analysis and visualization. The raw sequence files of control (Data file 1, Table 1) and Spd treated (Data file 2, Table 1) were deposited in NCBI, SRA database. 16S rRNA sequences were used to pick operational taxonomic units (OTUs) at 97% similarity threshold from the Greengenes database. To determine sampling depth and species richness, rarefaction curves were plotted (Data file 3, Table 1). The read details were mentioned in Data file 4, in Table 1. The taxonomic profile analysis of control and Spd fed gut tissues were indicated in stacked bar plots in Datafile 5 Table 1.

**Table 1:** Overview of *B. mori* gut metagenome data files/data sets available at https://www.ncbi.nlm.nih.gov/sra/?term=SRP126130

Label	Name of data file/data set	File types (file extension)	Data repository and identifier (DOI or accession number)
Data file 1	Mulberry silkworm gut metagenome	.fastq	https://www.ncbi.nlm.nih.gov/sra/SRX3444003[accn] [28]
Data file 2	Gut metagenome of Spermidine fed *Bombyx mori*	.fastq	https://www.ncbi.nlm.nih.gov/sra/SRX3989572[accn] [28]
Data file 3	Figure S1	.pdf	https://dx.doi.org/10.6084/m9.figshare.10565510
Data file 4	Additional file 1	.pdf	https://dx.doi.org/10.6084/m9.figshare.11663784
Data file 5	Additional file 2	.pdf	https://dx.doi.org/10.6084/m9.figshare.11664165

The *Proteobacteria* provide nutrients to their host, while *Firmicutes* increases the energy harvest from the diet [28]. Spd feeding in the mouse model increased the abundance of *Prevotella* and *Clostridium* [29]. Another major bacterial symbiont in Spd treated gut, *Halomonas* were reported to produce extracellular polysaccharides that help in adhesion and create microenvironments which favour, interaction and a cellular association between microorganisms [30].

## Limitations

Current data is based on only one biological replicate for single strain of *B. mori* gut metagenome sequencing.

## Data Availability

The data described here is available in the NCBI, SRA database under the accession (https://www.ncbi.nlm.nih.gov/sra/?term=SRP126130). Please refer data file 1 and 2 for links to the sequence data.

## Abbreviations

B.mori: Bombyx mori
Spd: Spermidine
